# Environmental Factors Promoting Neural Plasticity: Insights from Animal and Human Studies

**DOI:** 10.1155/2017/7219461

**Published:** 2017-06-14

**Authors:** Laura Mandolesi, Francesca Gelfo, Laura Serra, Simone Montuori, Arianna Polverino, Giuseppe Curcio, Giuseppe Sorrentino

**Affiliations:** ^1^Department of Movement Sciences and Wellbeing, University “Parthenope”, Via Medina 40, 80133 Naples, Italy; ^2^Laboratory of Experimental Neurophysiology and Behaviour, IRCCS Fondazione Santa Lucia, Via del Fosso di Fiorano 64, 00143 Rome, Italy; ^3^Department of Clinical and Behavioural Neurology, IRCCS Fondazione Santa Lucia, Via Ardeatina 306, 00179 Rome, Italy; ^4^Department of Systemic Medicine, University of Rome Tor Vergata, Via Montpellier 1, 00133 Rome, Italy; ^5^Neuroimaging Laboratory, IRCCS Fondazione Santa Lucia, Via Ardeatina 306, 00179 Rome, Italy; ^6^Istituto di Diagnosi e Cura Hermitage Capodimonte, Via Cupa delle Tozzole 2, 80131 Naples, Italy; ^7^Department of Life, Health and Environmental Sciences, University of L'Aquila, Piazz.le S. Tommasi, 1, 67100 L'Aquila, Italy

## Abstract

We do not all grow older in the same way. Some individuals have a cognitive decline earlier and faster than others who are older in years but cerebrally younger. This is particularly easy to verify in people who have maintained regular physical activity and healthy and cognitively stimulating lifestyle and even in the clinical field. There are patients with advanced neurodegeneration, such as Alzheimer's disease (AD), that, despite this, have mild cognitive impairment. What determines this interindividual difference? Certainly, it cannot be the result of only genetic factors. We are made in a certain manner and what we do acts on our brain. In fact, our genetic basis can be modulated, modified, and changed by our experiences such as education and life events; daily, by sleep schedules and habits; or also by dietary elements. And this can be seen as true even if our experiences are indirectly driven by our genetic basis. In this paper, we will review some current scientific research on how our experiences are able to modulate the structural organization of the brain and how a healthy lifestyle (regular physical activity, correct sleep hygiene, and healthy diet) appears to positively affect cognitive reserve.

## 1. Introduction

Numerous clinical and experimental studies demonstrated that many environmental factors may affect both the physiological functions of the central nervous system (CNS) and its ability to counteract pathological changes. It has been demonstrated that experience shapes our neural circuits, making them more functional, keeping them “young.” Experience is then the factor which induces our brain to be more plastic. In other words, experience may increase neuroplasticity. The complex of molecular and cellular processes known as neuroplasticity represents the biological basis of the so called “cerebral reserves.” The first to introduce the concept of “reserve” was Yaakov Stern who noticed a higher prevalence of Alzheimer's disease (AD) in people with lower education. For Stern, the reserve is a mechanism, which may explain how, in the face of neurodegenerative changes that are similar in nature and extent, individuals vary considerably in the severity of cognitive aging and clinical dementia [1]. Clinical studies provide evidence that people with a high level of education have a slower cognitive decline [2, 3].

According to Stern, two types of cerebral reserves are recognized: brain reserve (BR) and cognitive reserve (CR). BR is based on the protective potential of anatomical features such as brain size, neuronal density, and synaptic connectivity. This reserve is passive and is also defined as the amount of brain damage that can be sustained before reaching a threshold for clinical expression [1]. It also explains differential susceptibility to functional impairment in the presence of pathology or neurological insult [4]. This concept arose by the observation that the prevalence of dementia is lower in individuals with larger brains [5–7]. In contrast, CR posits the differences in cognitive processes as a function of lifetime intellectual activities and other environmental factors that explain the nonlinear relationship between the severity of patients' brain damage and the correspondent clinical symptoms. The CR suggests that the brain actively copes with brain damage by using the preexisting cognitive processes or by enlisting compensatory mechanisms [1, 3]. Thus, CR represents a functional reserve because it is based on the efficiency of neural circuits [8]. CR is considered an “active reserve” because the brain dynamically attempts to cope with brain damage by using preexisting cognitive processing networks or by enlisting compensatory networks [1, 3]. It is important to emphasize that BR and CR are not mutually exclusive but are involved together, at different levels, in providing protection against brain damage [9]. For this reason, it is possible to refer to the accumulated structural reserve (BR) and capacity for functional compensation (CR) using the new construct of “brain and cognitive reserve” (BCR) [10]. In fact, any morphological change results in a modification of the functional properties of a circuit and vice versa, and any change in neuronal efficiency and functionality is based on morphological modifications. For example, factors associated with an increased CR, such as cognitively stimulating experiences or a great deal of physical activity, are associated with neurogenesis, increased levels of neurotrophic factors, and diminution of neuronal apoptosis [11]. Therefore, functional and anatomical factors interact in the construction of the cerebral reserves [12].

In clinical research, we can study the relation between structural (BR) and functional (CR) changes by analyzing the gray matter damage in AD patients (structural measure) and then correlating it with a cognitive evaluation (functional measure) [13].

More direct measures of experience-due structural and functional changes are provided by experimental research on animal models. For example, BR measures are the changes at cellular and molecular levels [14], while direct CR measures are the performances in behavioral tasks, such as spatial tasks [8, 15]. The studies carried out by using enriched environment animal models enabled us to understand what kinds of experiences are necessary to trigger the phenomenon of brain plasticity and thus to increase cerebral reserves.

The purpose of the present work is to provide an up-to-date overview on the effects of the environmental factors on promoting neural plasticity in physiological and pathological conditions taking into account both human and animal studies.

## 2. Animal Studies

There is evidence showing that individuals with more CR are those who have a high level of education, who maintain regular physical activity, and who eat in a healthy way [16–19]. Despite such evidence, human studies do not allow us to determine whether one kind of experience determines the increase in cognitive reserve more than the other ones. Human research cannot separate the different variables that make up experience because we cannot analyze them separately. The experimental research on animals may compensate for these shortcomings by forcing the stimulation of a specific experience or a combination of experiences, as occurs in enriched environment animal models. The animal models of environmental enrichment (EE) allow us to obtain a direct, real, and tangible measure of which environmental factors are able to model neuronal circuits [8].

EE represents an experimental model in which the animal is exposed for a certain time period to a combination of experiences, such as an intense motor activity and sustained cognitive stimulation. This condition is usually compared to the standard condition of regular laboratory housing [20].

The majority of EE animal models concern rodents, but studies have also been carried out on nonhuman primates, birds, and fish [21].

**Figure 1:**
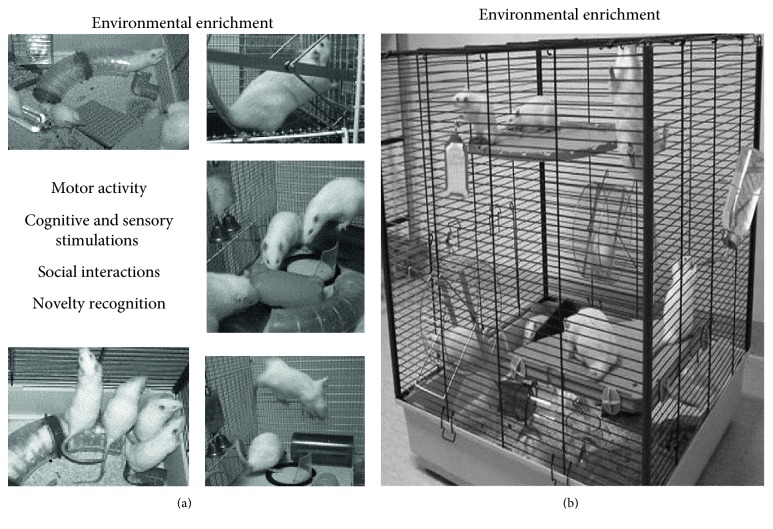
A typical enriched setting that enhances motor, sensory, cognitive, and social stimulations in rodents is illustrated in (b). In (a), the different components acting in the environmental enrichment are shown. Modified from [8].

At the first glance, it may seem strange that the EE in animals may be really compared to cognitive, motor, social, and emotional experiences in humans. Although this correlation may seem impossible, exposure of animals to an enriched environment is actually similar to that which occurs in human lifestyle [8]. In fact, in humans, the development of reserves can be influenced by several factors, such as educational level, physical activity, social integration, and emotional involvement. In animal models, all these factors are provided by the environmental complexity and novelty the animals are exposed to. The repeated replacing of objects in the home cages creates a wide range of opportunities for enhanced cognitive stimulation, formation of efficient spatial maps, and heightened ability to detect novelty. Physical training is represented by foraging in large cages, exploration of new objects that are constantly introduced into the cages, and general motor activity related to the use of wheels. The social aspect that characterizes human relationships may be mimicked by rearing the animals in a group of conspecifics. In fact, if the animals are stimulated to live together in the same cage, a social hierarchy emerges and a dominant figure arranges and controls the spaces of the cage and when to eat.  Figure 1 shows an example of the rearing in an enriched environment.

The first to introduce the experimental concept of enriched environment was Donald Hebb, although it was the famous American psychologist Mark Richard Rosenzweig who clarified the enriched environment as “a combination of complex inanimate and social stimulations” [22].

Thus, the implementation of a setting of EE is a quite complex procedure, in which motor activity, cognitive abilities, and social interaction should be taken into account. Although recently it has been shown that also physical activity alone is able to increase CR, most studies show that all these factors should be stimulated to increase brain plasticity [8].

An EE paradigm is used with healthy animals to analyze neuroplastic functional and structural changes [23], with animals that present neurodegenerative lesions or transgenic mutations to analyze neuroprotective and therapeutic effects [10, 15, 24–27], and recently even with an animal model of psychiatric disorders, such as schizophrenia, to evaluate the ameliorative effects on behavioral symptoms [28–30].

In general, cognitive abilities in animals are evaluated by means of specific behavioral tasks such as Morris water maze (MWM) and radial arm maze (RAM) that analyze the different facets of spatial memory. In fact, the memory can be divided into at least two types, such as declarative and procedural. Declarative knowledge refers to things that we know that are accessible to conscious recollection (“knowing that”), while procedural material regards memories on how to do something (“knowing how”) and those that are seen as implicit and unconsciously learned [31]. The two types of memory have different and specific neural correlates. Declarative memory mainly involves the hippocampal structures, while procedural learning and memory rely more on the cerebellum and basal ganglia [32–34]. Majority results discussed in the next sessions come from MWM and RAM behavioral tasks.

### 2.1. Functional and Structural Effects of EE

**Figure 2:**
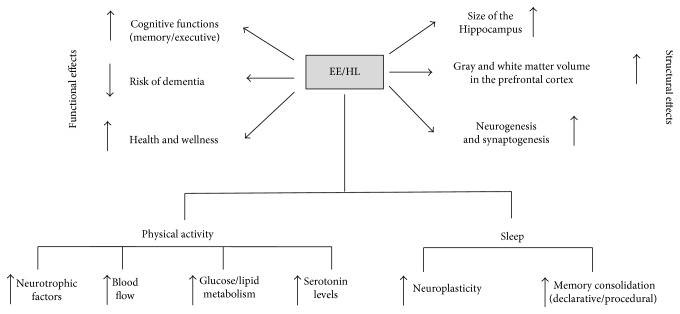
Schematic representation of the structural and functional effects of environmental enrichment (EE) on animal models and healthy lifestyle (HL) on humans.

Many studies conducted on healthy animals show that rearing in an enriched environment has significant functional and structural effects (Table 1, Figure 2).

To evaluate the functional effects of EE on the performances in behavioral tasks, spatial tasks are analyzed. In particular, these tasks permit us to analyze the different facets of spatial cognitive function and then to evaluate the functioning of underlying neural circuits. For example, Leggio and coworkers compared the spatial performances in radial arm maze and in Morris water maze of healthy animals reared in an enriched environment for three months after the weaning with those of animals reared in standard conditions [23]. In both spatial tasks, the animals reared in an enriched environment made fewer errors than the conspecifics reared in standard laboratory conditions and showed a precocious development of spatial cognitive mapping of the environment.

In EE structural effects, the changes at cellular level (such as neurogenesis, gliogenesis, angiogenesis, and synaptogenesis) and the alterations at molecular level (such as changes in neurotransmitter and neurotrophin expression) are considered [15]. By studying synaptogenesis, Gelfo and coworkers evidenced as indices of improved neuronal circuitry the increased dendritic length and spine density shown by the frontal and parietal pyramidal neuron apical and basal arborizations of rats reared in EE [35]. Molecular effects that follow EE have been demonstrated by analyzing the neurotrophin levels in brain structures where neurotrophins are produced or transported. In particular, multiple studies in rodent models showed that EE increases the expression of a brain-derived neurotrophic factor (BDNF) in the hippocampus that heavily supports the EE-induced improvement in learning and memory [36, 37]. Moreover, neurotrophin levels were found to be also increased in the cerebellum and other cerebral areas following EE [14].

Functional and structural effects of EE are analyzed even from a transgenerational point of view. In particular, Caporali and coworkers [38] reared female rats in enriched conditions and then studied the motor behavior and the neurotrophin levels of their pups reared in standard conditions. This study demonstrates that positive maternal experiences were transgenerationally transmitted and influenced offspring phenotype at both behavioral and biochemical levels. In fact, the pups from enriched mothers acquired complex motor behaviors earlier than the pups from mothers reared in standard conditions. Moreover, in the pups from enriched mothers, the cerebellar and striatal neurotrophin expression was significantly higher. Evidence presents that also paternal EE is able to transgenerationally alter affective behavioral and neuroendocrine phenotypes of the offspring [39–41]. These studies suggest that the cerebral reserves could be even inherited.

### 2.2. Neuroprotective Effects of EE

As we mentioned, many studies showed that EE or even just motor exercise induces neuroprotection against neurodegenerative diseases [15, 24–26]. In a brilliant review on the EE models, Nithianantharajah and Hannan showed that motor exercise alone produces a positive effect at behavioral, cellular, and molecular levels on some diseases that affect the cognitive-motor sphere, such as Huntington's disease (HD), Parkinson's disease (PD), and AD [15]. To give some examples, in HD mouse models, it was demonstrated that wheel-running exercise delays the onset of specific motor deficits [42–44] and diminished the impairment in spatial memory and cognitive flexibility, also attenuating neuropathology [45]. Behavioral performance has been demonstrated to be improved by physical training also in PD rodent models [46, 47], with neuroprotective effects on the regulation of neurochemical factors [48, 49]. Finally, in AD, an intensive locomotor training increases the quality of performance in behavioral tasks concerning spatial learning and memory [50]. At cellular level, a decrease in beta-amyloid plaques occurs and, only in the case of more complex stimulation, an increase in the levels of neurotrophic substances as synaptophysin was also observed [51–53].

Examples coming from transgenic murine models, which provide the precious advantage to determine exactly when a structural alteration occurs, allow to evaluate when it is best to enrich the animals. For example, by means of transgenic AD mice (Tg2576), Verret and coworkers showed that the EE effects are more powerful if the animals are reared in an enriched environment before the formation of beta-amyloid plaques [54], that is, before their deleterious effects on brain function and memory processing become permanent.

Decreased levels of beta-amyloid plaque in response to EE have been highlighted also by Beauquis and coworkers who analyzed the astroglial changes in the hippocampus of transgenic animals [55]. In fact, growing evidence shows that glial changes may precede neuronal alterations and behavioral impairment in the progression of AD and that the modulation of these changes could be addressed as a potential therapeutic strategy [56–58]. In particular, Beauquis and coworkers evidenced that in enriched transgenic animals (APP mice), a decrement in levels of astrocytes was present, suggesting that glial alterations have an early onset in AD pathogenesis and the exposure to an enriched environment is an appropriate strategy to reverse them.

Moreover, the confirmation that glial alterations play an important role in cerebral reserves comes from a recent study that investigated the functional and structural effects of intermittent EE (3 hours/day for two months) on aged rats [58]. In fact, even at advanced ages, behavioral results showed that EE improved performances in a radial water maze task and structural data evidenced plastic changes in the hippocampal astrocytes suggesting that these neuroplastic alterations are involved in a coping mechanism with age-related cognitive impairment.

Several authors wondered until which point in life the enrichment has positive effects on cognitive function. Fuchs and coworkers assessed the impact of late housing condition (e.g., from the age of 18 months) on spatial learning and memory of aged rats (24 months) previously exposed or unexposed to EE during young adulthood (until 18 months) [59]. The results showed that late EE was not required for spatial memory maintenance in aged rats previously housed in EE. In contrast, late EE mitigates spatial memory deficit in aged rats previously unexposed to EE. These outcomes suggest that EE exposure up to middle age provides a reserve-like advantage that supports an enduring preservation of spatial capabilities in old age [60, 61].

In addition to the transgenic animal models of EE, also the studies on lesioned animals contributed to highlighting the neuroprotective role of environmental stimulation. For example, it was found that rats exposed to EE at weaning about three months before a cholinergic basal forebrain depletion (which mimics AD) recover some cognitive abilities such as spatial memory and cognitive flexibility [62]. These improvements in the cognitive-motor domain were also accompanied by changes at the morphological level [26], demonstrating once again the close link between structure and function and, in this case, between CR and BR.

The main neuroprotective effects of EE are shown in Table 1.

## 3. Environmental Factors and Lifestyle in Human

Research on animal models provides an important insight into understanding the key role of environmental factors in promoting cognitive reserve. On the other hand, human studies showed that not only high-demand cognitive activities are able to improve cognitive skills and counteract a physiological and pathological cognitive decline but even other environmental factors such as regular physical activity and correct sleep hygiene can substantially contribute to brain well-being.

### 3.1. Physical Activity (PA) and Neuroplasticity

In EE animal models, it has been shown that motor exercise has significant effects on neuroplasticity and counteracts a pathological cognitive decline [10, 15]. In humans, it seems necessary to distinguish between physical activity (PA) and physical exercise (PE). In fact, PA is any movement of the body produced by skeletal muscles that results in energy expenditure over the baseline levels, including all structured daily activities, such as housework and leisure activities. Conversely, PE is a structured and repetitive physical activity, aimed at maintaining or improving one or more components of physical fitness.

PA and PE are often related to health benefits in the prevention and in the treatment of many pathological conditions, such as metabolic diseases [63–65] as well as diseases associated with compromised cognition and brain function [66]. Several studies do exist showing that the practice of regular and constant PA reduces the risk of developing dementia [67].

PA increases blood flow, improves cerebrovascular health, and determines benefits of glucose and lipid metabolism carrying “food” to the brain. It has been showed that PA causes neural plasticity phenomena. For example, PA facilitates the release of neurotrophic factors like BDNF, stimulates neurogenesis phenomena, and determines structural changes such as the improvement of white matter integrity [68]. The brain changes are inevitably reflected in functional modifications. In this context, children with higher levels of aerobic fitness showed greater brain volumes in gray matter brain regions (structural changes) and the best performances in learning and memory tasks (functional changes) in comparison to sedentary children [69]. It is important to underlie that all the structural and functional changes are derived by an aerobic type of PA. Recently, it has been showed that only regular aerobic exercise is associated with larger size of the hippocampal regions [70]. Moreover, aerobic exercise increases gray and white matter volume in the prefrontal cortex [71] and increases the functioning of key nodes in the executive control network [72, 73] (Figure 2).

### 3.2. Sleep and Neuroplasticity

In the last decades, it has been shown that sleep is an essential feature of animal and human brain plasticity, which involves both basic (e.g., [74]) and higher-order functions (e.g., [75]).

Sleep is an active, repetitive, and reversible behavior that is in the service of several different functions that occur all over the brain and the body [76, 77]: from repair and growth to learning or memory consolidation and up to restorative processes. This basic role of sleep is also indirectly substantiated by the fact that almost all the animal species, from fruit flies to the biggest mammals [78], share a behavioral state that can be defined as “sleeplike.” Thus, if sleep subserves all these aspects of animal life, it would be seen as a crucial survival-directed drive, so that chronic or repeated sleep deprivation in rodents brings cellular and molecular changes in the brain [79] while in humans, it can dramatically disrupt several high-order cognitive functions [75, 80–83].

Different hypotheses have been suggested to deeply explain the functions of sleep, and one of the well-accepted ideas is that sleep is linked to memory, learning, and neuroplasticity mechanisms [74] (Figure 2).

Several studies showed that sleep plays an important role in learning processes and memory consolidation [84, 85] although no direct relationships have been found between different kinds of memory and different sleep stages [86]. These studies clearly indicated that sleep deprivation can impair learning and different kinds of memory that can be divided into at least two types, such as declarative and procedural (as discussed above). Thanks to this distinction, a dual-process hypothesis has been proposed [87]: the effect of a sleep state on memory processes would be task-dependent, with the procedural memory gaining from REM (rapid eye movement) sleep and declarative memory from NREM (nonrapid eye movement) sleep [88].

But other data [89] have been interpreted as in line with the alternative point of view, that is, the hypothesis of a sequential processing of memories during sleep stages [90, 91] suggesting that memory formation would be prompted by NREM sleep (and particularly by its slow-wave content, namely, stages 3 and 4) and then consolidated by REM sleep, indicating that for an efficient consolidation of both knowledge (declarative) and skills (procedural), the worst enemy is sleep loss or, at least, sleep fragmentation.

The nature of the link between sleep and synaptic plasticity is not fully understood: several different processes of synaptic reorganization would occur during sleep period, but their functional role needs to be clarified. In a very recent review [74], it has been discussed that induction of plastic changes during wake can produce coherent and topographically specific local changes in EEG slow activity in the subsequent sleep and that during sleep, synaptic plasticity would be restored.

Independently by the actual nature of the link between sleep and neuroplasticity, now, it is well known and accepted that a good quality of sleep allows an efficient and successful aging [92]. In fact, several recent studies have clearly indicated the relevance of sleep quantity and quality as a marker of general health, well-being, and adaptability in later life [93–95]. This literature can help in developing health programs devoted to the oldest aim of improving sleep hygiene in order to guarantee avoidance of disease, maintenance of high cognitive and physical function, and continued engagement with life.

## 4. Conclusions

Experimental research strongly suggests that in order to increase our cerebral reserves, we have to follow a lifestyle that takes into account many factors. Clinical studies provided evidence that individuals with more cerebral reserves are those who have a high level of education, who maintain regular physical activity, who eat in a healthy way, and so on. The EE animal models confirmed that the experience plays a key role in increasing brain plasticity phenomena. Although we are still far from identifying the basic ingredient responsible for increasing our brain plasticity and for counteracting neurodegenerative damage, we can say with confidence that to deal with physiological and pathological situations, it is not only important to be “genetically lucky” but also to maintain a lifestyle rich in experiences also including high levels of physical activity and good sleep hygiene.

## Figures and Tables

**Table 1:** Structural and functional effects of environmental enrichment (EE).

Sample		EE condition	Functional Effects (behavioral effects)	Structural Effects (molecular and cellular effects)	Refs.
Effects of EE on healthy rodents					
Wistar rats		EE from weaning for 2.5/3 months	Precocious development of spatial cognitive map; enhanced spatial memory and cognitive flexibility	Increases dendritic length and spine density in frontal and parietal pyramidal neuron apical and basal arborizations; synaptogenesis; increases of BDNF levels in the hippocampus and cerebellum	[14, 23, 35–37, 40]
Wistar rats C57BL/6J mice		Maternal and paternal EE: a transgenerational model	In pups: accelerated acquisition of complex motor behaviors; decreased anxiety-related behaviors	In pups: high expression of neurotrophin in cerebellar and striatal areas; low ACTH levels	[38–41]

Neuroprotective effects of EE on neurodegeneration					
Neurodegenerative disorders	HD mouse models	Running exercise about from 4 weeks of age	Partially delayed onset of motor symptoms and cognitive deficits (memory/executive functions)	Altered BDNF mRNA levels	[10, 15, 42–45]
		EE about from 4 weeks of age	Delayed onset of motor symptoms and cognitive deficits (memory/executive functions)	Decreased cortical and striatal volume loss; ameliorated deficit in neurogenesis; increased neurotrophin expression; enhanced CB1 receptor levels	
	PD mouse models	Running exercise from 6 weeks	Attenuated motor impairment, reduced anxiety behavior	Decreased loss of striatal DA	[10, 15, 46–49]
	AD mouse/rat models	Intensive locomotor training	Increases performances in spatial memory tasks	Decreased beta-amyloid plaques	[10, 15, 24–27, 50–57, 62]
		EE from weaning for 2.5/3 months; EE for 2 months at different age	Enhanced spatial memory and executive functions (cognitive flexibility)	Decreased beta-amyloid plaques; increased levels of neurotrophic substances; increased spine number and density in pyramidal neurons	
Aging		EE and locomotor training in middle age	Preservation of spatial abilities in old age	Changes in hippocampal astrocytes; hippocampal neurogenesis	[58–61]

EE refers to a complex stimulation of experiences. BDNF: brain-derived neurotrophic factor; NGF: nerve growth factor; ACTH: adrenocorticotropic hormone; HD: huntington's disease; PD: parkinson's disease; AD: alzheimer's disease; DA: dopamine.
